# Ectopic *GRHL2* Expression Due to Non-coding Mutations Promotes Cell State Transition and Causes Posterior Polymorphous Corneal Dystrophy 4

**DOI:** 10.1016/j.ajhg.2018.02.002

**Published:** 2018-03-01

**Authors:** Petra Liskova, Lubica Dudakova, Cerys J. Evans, Karla E. Rojas Lopez, Nikolas Pontikos, Dimitra Athanasiou, Hodan Jama, Josef Sach, Pavlina Skalicka, Viktor Stranecky, Stanislav Kmoch, Caroline Thaung, Martin Filipec, Michael E. Cheetham, Alice E. Davidson, Stephen J. Tuft, Alison J. Hardcastle

**Affiliations:** 1 Research Unit for Rare Diseases, Department of Paediatrics and Adolescent Medicine, First Faculty of Medicine, Charles University and General University Hospital in Prague, Ke Karlovu 2, Prague 128 08, Czech Republic; 2 Department of Ophthalmology, First Faculty of Medicine, Charles University and General University Hospital in Prague, U Nemocnice 2, Prague 128 08, Czech Republic; 3 UCL Institute of Ophthalmology, University College London, London EC1V 9EL, UK; 4 Institute of Pathology, Third Faculty of Medicine, Charles University, Faculty Hospital Kralovske Vinohrady, Srobarova 50, Prague 100 34, Czech Republic; 5 Moorfields Eye Hospital, London EC1V 2PD, UK

**Keywords:** non-coding mutation, regulatory region, ectopic expression, GRHL2, epithelial-to-mesenchymal transition, mesenchymal-to-epithelial transition, PPCD, corneal dystrophy, corneal edema, corneal endothelium

## Abstract

In a large family of Czech origin, we mapped a locus for an autosomal-dominant corneal endothelial dystrophy, posterior polymorphous corneal dystrophy 4 (PPCD4), to 8q22.3–q24.12. Whole-genome sequencing identified a unique variant (c.20+544G>T) in this locus, within an intronic regulatory region of *GRHL2*. Targeted sequencing identified the same variant in three additional previously unsolved PPCD-affected families, including a *de novo* occurrence that suggests this is a recurrent mutation. Two further unique variants were identified in intron 1 of *GRHL2* (c.20+257delT and c.20+133delA) in unrelated PPCD-affected families. GRHL2 is a transcription factor that suppresses epithelial-to-mesenchymal transition (EMT) and is a direct transcriptional repressor of *ZEB1. ZEB1* mutations leading to haploinsufficiency cause PPCD3. We previously identified promoter mutations in *OVOL2*, a gene not normally expressed in the corneal endothelium, as the cause of PPCD1. OVOL2 drives mesenchymal-to-epithelial transition (MET) by directly inhibiting EMT-inducing transcription factors, such as *ZEB1.* Here, we demonstrate that the *GRHL2* regulatory variants identified in PPCD4-affected individuals induce increased transcriptional activity *in vitro*. Furthermore, although *GRHL2* is not expressed in corneal endothelial cells in control tissue, we detected GRHL2 in the corneal “endothelium” in PPCD4 tissue. These cells were also positive for epithelial markers E-Cadherin and Cytokeratin 7, indicating they have transitioned to an epithelial-like cell type. We suggest that mutations inducing MET within the corneal endothelium are a convergent pathogenic mechanism leading to dysfunction of the endothelial barrier and disease.

## Introduction

Posterior polymorphous corneal dystrophy (PPCD) is a rare autosomal-dominant disorder, primarily affecting the corneal endothelium and Descemet membrane. The severity and phenotype of PPCD is variable.^1^ Mild manifestations of the disease include asymptomatic corneal endothelial changes such as vesicular, band-like, and geographic lesions. In severe cases, corneal endothelial failure may occur and corneal transplantation is required to restore vision.^1^, ^2^, ^3^, ^4^ Aberrant corneal endothelial cells have been shown to proliferate and migrate onto the trabecular meshwork and iris acquiring an epithelial-like morphology.^1^, ^5^, ^6^, ^7^, ^8^ Secondary complications are common and include corneal edema, glaucoma, iris adherence to the cornea, and corectopia.^1^, ^2^

PPCD is a genetically heterogeneous condition, with approximately a third of cases attributed to heterozygous mutations in the transcription factor encoding gene *ZEB1* (MIM: 189909) (PPCD3 [MIM: 609141]).^3^, ^9^, ^10^, ^11^ Recently, we and others have established that heterozygous regulatory mutations in the promoter of *OVOL2* (MIM: 616441) cause PPCD1 (MIM: 122000).^2^, ^12^ ZEB1 and OVOL2 control cell state, through regulation of epithelial-to-mesenchymal transition (EMT) and the converse process of mesenchymal-to-epithelial transition (MET), through a mutually inhibitory pathway.^13^, ^14^ EMT and MET are central processes in development, and these finely tuned and reversible cell state transition pathways also support the maintenance of cellular identity and function.^15^, ^16^ Aberrant regulation of MET and EMT underpins tumor progression and malignant transformation processes, as well as playing an important role in other disease conditions including fibrosis, wound repair, and inflammation.^17^, ^18^

Corneal endothelial cells are embryonically derived from the neural crest and form a monolayer of post-mitotic hexagonal cells on the inner surface of the cornea. They are specialized cells that have a barrier-pump function, governing fluid and solute transport across the posterior surface of the cornea and maintaining the cornea in a relatively dehydrated state that is essential for optical transparency.^19^, ^20^ Haploinsufficiency and subsequent reduced expression of *ZEB1* in the corneal endothelium is thought to underlie the pathology of PPCD3,^10^ whereas inappropriate ectopic expression of *OVOL2* in corneal endothelial cells is the proposed mechanism for PPCD1.^2^, ^12^ The disrupted balance of cell state transition regulators OVOL2 and ZEB1 within the diseased corneal endothelial cells could result in cellular *trans*-differentiation of the corneal endothelial cells into an epithelial-like state through the MET pathway.^2^, ^10^, ^14^, ^21^, ^22^ This hypothesis is supported by multiple studies demonstrating the epithelial-like phenotype of endothelial cells in PPCD, including histopathological and transcriptomic studies,^1^, ^5^, ^6^, ^21^, ^22^ and is likely a consequence of gene mutations specifically affecting corneal endothelial cells.

Despite these recent advances in our understanding of the molecular basis of PPCD, there is evidence for further genetic heterogeneity of PPCD.^12^, ^21^, ^23^ Here, we describe an additional PPCD locus, PPCD4, which was mapped to 8q22.3–q24.12, and the subsequent identification of causative non-coding variants in *GRHL2* with further evidence for the importance of MET in PPCD.

## Material and Methods

### Study Subjects and Clinical Examination

All participants signed informed consent approved by the ethics committee of the General University Hospital in Prague (reference no. 151/11 S-IV) or Moorfields Eye Hospital (REC references 13/LO/1084 and 09/H0724/25) before inclusion in the study. Ophthalmic examination included best corrected distance Snellen visual acuity (BCVA) converted to decimal values, intraocular pressure, slit-lamp biomicroscopy and specular microscopy (Noncon ROBO Pachy SP-9000; Konan Medical Inc.) and spectral-domain optical coherence tomography (SD-OCT) (Spectralis; Heidelberg Engineering GmbH). Genomic DNA was extracted from venous blood samples using a Gentra Puregene blood kit (QIAGEN) or from saliva using a Oragene kit (Oragene OG-300, DNA Genotek).

### Linkage Analysis

**Figure 1:**
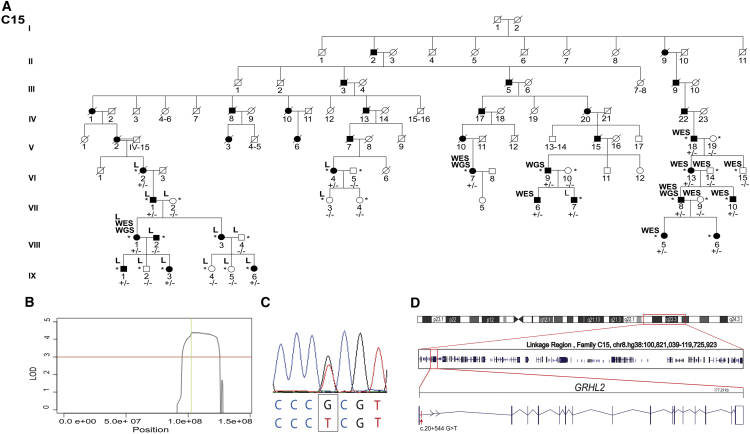
Identification of a Locus for Autosomal-Dominant PPCD on 8q22.3–q24.12 and a Unique Variant in Intron 1 of *GRHL2* (A) Abridged pedigree structure of PPCD-affected family C15 of Czech origin. L indicates samples used for SNP genotyping and linkage analysis. WES and WGS indicate DNA samples analyzed by whole-exome and whole-genome sequencing, respectively. Individuals who were heterozygous for the *GRHL2* c.20+544G>T mutation are indicated by +/−, and those lacking the mutation are indicated by −/−. (B) Linkage analysis identified a single locus with a significant LOD score (>3, red line) spanning chromosome 8q22.3–q24.12 from chr8.hg38:100,821,039–119,725,923 with a maximum LOD score of 4.38 (green line). (C) Heterozygous variant c.20+544G>T (chr8.hg38:101,493,333G>T) identified by WGS, located in intron 1 of *GRHL2* (GenBank: NM_024915; Ensembl: ENST00000251808.7), was confirmed by Sanger sequencing. (D) Boxed region on chr8 depicts the PPCD4 linked interval, and the position and exon structure of the *GRHL2* gene (5′ to 3′) and intronic mutation are shown.

Linkage analysis was performed using selected individuals from family C15 (Figure 1A). Nine affected (VI:2, VI:4, VII:1, VIII:1, VIII:3, VII:7, IX:1, IX:3, IX:6) and seven unaffected samples (VII:2, VII:3, VIII:2, VIII:4, IX:2, IX:4, IX:5) were genotyped using an Illumina Omni2.5 Exome-8 array. Parametric linkage analysis, assuming dominant inheritance of a fully penetrant rare allele (disease allele frequency 0.00001) was performed using MERLIN.^24^ The following criteria were applied to select markers for linkage: only polymorphic SNPs with annotated rs numbers were analyzed, Mendelian inconsistent SNPs or SNPs with missing alleles were discarded, a SNP density of 0.1 cM was maintained.

### Whole-Exome Sequencing (WES)

WES was performed using a TruSeq exome enrichment kit (Illumina) and Illumina HiSeq2000 sequence platform on DNA samples of affected individuals VIII:1, VI:7, VII:6, and VII:10 from family C15, and using a SureSelect Human All Exon 50Mb Kit (Agilent) and Illumina HiSeq2000 sequence platform for individuals V:18, VI:13, VI:14. VI:15, VII:8, VII:9, and VIII:5. Reads were aligned to the GRCh38/hg38 human reference sequence with Novoalign v.2.05 (Novocraft). The WES data were analyzed using the Phenopolis platform.^25^ ExomeDepth was used to identify copy-number variants (CNVs).^26^ Aligned data were visualized with the Integrated Genomics Viewer (IGV, Broad Institute). On the basis that PPCD is a rare dominant disease, WES data were filtered for rare variants with a minor allele frequency (MAF ≤ 0.005 according to ExAC) in family C15 and a control WES dataset generated from 20 unrelated individuals of Czech origin (National Centre for Medical Genomics).

### Whole-Genome Sequencing (WGS)

Four distantly related affected individuals (VI:7, VI:9, VII:8, and VIII:1) from C15 were analyzed by WGS using a TruSeq Nano DNA library preparation kit and a HiSeq X Ten sequencer (Illumina). Reads were aligned to the GRCh38/hg38 human reference sequence with Novoalign v.2.05 (Novocraft). Variant calling was performed with GATK HaplotypeCaller^27^ and annotated using the Variant Effect Predictor (VEP)^28^ which provides allele frequency annotation in various control datasets, predicts the effects of variants on nearby transcripts, and reports the potential regulatory role for non-coding regions.

### Sanger Sequencing of Potential Regulatory Regions of *GRHL2*

For unsolved PPCD-affected case subjects, a region of 2,728 bp (chr8 hg38:101,491,361–101,494,128) encompassing potential regulatory regions of *GRHL2*, including the 5′ untranslated region (UTR), exon 1, and partial intron 1 (Figure S1), was amplified by PCR (GoTaqGreen, Promega, primers and conditions available on request) and Sanger sequenced using BigDye terminator sequencing on an ABI PRISM 3100 Genetic Analyzer (Applied Biosystems). *GRHL2* variants associated with disease have been submitted to ClinVar.

### *In Silico* Analysis of Variants

In addition to the annotation data provided by the VEP, variants of interest were also analyzed by splice site prediction tools Human Splicing Finder,^29^ NNSPLICE,^30^ MaxEntScan,^31^ and NetGene2.^32^ ENCODE (Encyclopedia of DNA elements) data were manually interrogated using IGV for transcription factor binding in the genomic region of interest containing candidate variants.^33^ The effect of variant on transcription factor binding was predicted by Alibaba 2.1^34^ and MatInspector.^35^ Alibaba 2.1 predicts transcription factor binding sites in an input nucleotide sequence using binding sites collected from TRANSFAC.^36^ MatInspector predicts transcription factor binding sites using a library of weight matrices.

### Cell Culture, RNA Extraction, and RT-PCR

RNA was extracted from whole corneal buttons donated after enucleation surgery for posterior segment melanoma and cell cultures, using an RNeasy Extraction Kit (QIAGEN). Primary endothelial cells were expanded and cultured as previously described.^2^ An immortalized human corneal endothelial cell line, B4G12, was cultured according to published protocols.^37^ Normal donor corneoscleral rims stored in Optisol (Chrion Ophthalmics) were obtained from Moorfields Lions Eye Bank, and limbal epithelial stem cells (HLEC/HLE-S) and stromal fibroblasts (SF) were isolated and cultured as previously described.^38^ HEK293 cells were cultured with standard reagents and conditions. cDNA was reverse transcribed using oligo(dT) priming with a Tetro cDNA synthesis kit (Bioline). *GRHL2* was amplified with intron-spanning primers from exon 4 to exon 8 forward 5′-GCGCCTATCTCAAAGACGAC-3′ and reverse 5′-CGTCCCAGGTAAAGGAAACA-3′ and beta actin was amplified using primers forward 5′-CTGGGACGACATGGAGAAAA-3′ and reverse 5′-AAGGAAGGCTGGAAGAGTGC-3′.

### Histology and Immunostaining

Cornea tissue from individual III:1 (age 8.5 years) from family C23 removed during penetrating keratoplasty was fixed in 10% neutral-buffered formalin. The sample was then processed into paraffin wax and 5-μm sections were cut. Sections were stained with tinctorial haematoxylin and eosin (H&E) using conventional methods.

A second cornea removed from individual II:1 (age 41 years) from family C23, also during penetrating keratoplasty, and a control cornea (Miracles In Sight Inc.) were embedded in optimal cutting temperature compound and snap frozen. Tissue sections were then cut to 4-μm thickness using a cryostat, thaw-mounted onto histological slides, and air-dried for 30 min. Immunostaining was performed manually using the Bond Polymer Refine Detection kit (DS9800, Leica). Sections were fixed for 10 min in acetone followed by 10 min in methanol. After washing with distilled water, a peroxidase block was used for 30 min to quench any endogenous peroxidase activity, followed by three washes with Tris-based saline 0.1% (v/v) Tween (TBS-T). Immunodetection of proteins of interest was carried out with the following primary antibodies: rabbit anti-GRHL2 (1:100, HPA004820, Sigma Aldrich), rabbit anti-N-cadherin (1:300, ab18203, Abcam), mouse anti-E-Cadherin (1:200, M3612, Dako), and human anti-Cytokeratin 7 (CK7, 1:2,000, M7018, Dako) for 1 hr at 37°C. Subsequently tissue sections were washed three times with TBS-T and incubated with post-primary linker IgG for 15 min for localization of mouse antibodies followed by three washes with TBS-T and incubation with poly-HRP IgG for 30 min for localization of rabbit antibodies. After three washes with TBS-T and distilled water, staining was visualized with 3,3′-diaminobenzidine tetrahydrochloride hydrate (DAB), washed, and counterstained with Mayer’s Haematoxylin to allow the visualization of nuclei. Tissue sections were then dehydrated in graded ethanol and in xylene prior to mounting with DPX mounting medium.

H&E staining was performed using a Leica Autostainer XL with integrated coverlipper (CV5030). Staining was visualized using a Nikon Eclipse 80i microscope equipped with a DXM1200C digital camera. Images of corneal sections were taken using the same magnification between the control and diseased tissue.

### Luciferase Assay

Primers were designed to amplify a genomic region that encompasses potential *GRHL2* regulatory regions, spanning all variants of interest. A 2,728-bp product (chr8:101,491,361–101,494,128) containing upstream sequence, exon 1, and partial intron 1 of the *GRHL2* gene (Figure S1) was amplified from control genomic DNA, cloned into pGEM-TEasy (Promega) and sub-cloned into the promoter-less firefly luciferase reporter vector pGL3-Basic (Promega). Primers used for cloning incorporated KpnI and *Nhe*I restriction site to facilitate subcloning (forward 5′-GGTACCCAAGCTTTCCACGTCCTCC-3′ and reverse 5′-GCTAGCCAAAGTTACCGGGGAAAGCAA-3′). Variants identified in PPCD4-affected individuals were introduced by site-directed mutagenesis using a Q5 Site-Directed Mutagenesis Kit (New England Biolabs) and all constructs were verified by Sanger sequencing. Wild-type or mutant *GRHL2* promoter pGL3-Basic plasmids (90 ng) were used to co-transfect HEK293 cells with 10 ng of pRL-CMV (CMV-promoter driven *Renilla* luciferase reporter, Promega) using TransIT-LT1 transfection reagent (Mirus). At 24 hr post-transfection, luciferase activity was measured using an Orion L Microplate Luminometer (Titertek Berthol) and a dual-luciferase reporter assay system (Dual-Glo Luciferase Assay System, Promega).

## Results

### Defining a New Locus for Autosomal-Dominant Posterior Polymorphous Corneal Dystrophy (PPCD4)

In a large autosomal-dominant PPCD-affected family of Czech origin (C15, Figure 1A), targeted Sanger sequencing did not identify any likely disease-associated variants within established PPCD-associated genes, including the *OVOL2* promoter region.^11^, ^23^ Furthermore, quantitative real-time PCR and Illumina HumanOmniExpress BeadChip SNP array analysis did not detect CNVs encompassing known PPCD-associated genes.^10^ Therefore, we performed WES using DNA samples from affected (VIII:1, VI:7, VII:6, VII:10, V:18, VI:13, VII:8, VIII:5) and unaffected (VI:14, VII:9, VI:15) individuals from family C15. WES data were filtered for rare variants in affected individuals that were absent from unaffected individuals; no potential mutations were identified. We therefore considered that an additional PPCD locus and/or a variant not captured by WES might be causative in this family.

We therefore defined the locus segregating with disease in family C15 through linkage analysis by genotyping nine affected and seven unaffected individuals from a large branch of family C15 (Figure 1A). A single locus was identified, chr8. hg38:100,821,039–119,725,923, spanning chromosome 8q22.3–q24.12, with a maximum LOD score of 4.38 (Figure 1B), thereby delineating a locus for PPCD (PPCD4).

### Identification of a Rare Non-coding *GRHL2* Variant in the Index PPCD4-Affected Family

Next, we performed WGS in four distantly related affected individuals from family C15 (Figure 1A) and filtered for variants located within the PPCD4 locus (chr8.hg38:100,821,039–119,725,923) that were shared between all four affected individuals. We filtered our WGS datasets to exclude all variants that have a MAF ≥ 0.005 in the gnomAD, Kaviar, 1000G, GoNL, and UK10X datasets. Using this approach, three unique variants were identified in the linkage region that were confirmed by Sanger sequencing (Table 1). Two variants were intergenic, and one variant occurred within intron 1 of *GRHL2*. We found no bioinformatic evidence to implicate the intergenic variants in regions of active promoters or enhancers. In contrast, c.20+544G>T (chr8.hg38:101,493,333G>T), located in intron 1 of *GRHL2*, maps to a promoter region for this gene (ENSR00000228091), reflected in the CADD score (Figures 1C and 1D; Table 1).

**Table 1:** Unique Variants within the PPCD4 Locus, chr8.hg38:100,821,039–119,725,923, Identified by WGS in Family C15

								**Allele Count/Total Alleles Screened**				
**Variant No.**	**Coordinates (hg19)**	**Coordinates (hg38)**	**Reference Allele**	**Observed Allele**	**Location**	**Closest Transcript**	**CADD score**	**Kaviar**	**gnomAD**	**1000G**	**UK10K**	**GoNL**
1	102,121,864	101,109,636	C	T	intergenic	*–*	7.875	0/26,378	0/30,978	0/5,007	0/7,562	0/998
2	102,505,561	101,493,333	G	T	intronic	*GRHL2*	10.76	0/26,378	0/30,978	0/5,007	0/7,562	0/998
3	115,648,021	114,635,792	A	T	intergenic	*–*	6.33	0/26,378	0/30,978	0/5,007	0/7,562	0/998

Three novel variants were identified within the mapped PPCD4 locus from four WGS datasets (C15; VI:7, VI:9, VII:8, VIII:1) filtered by (1) removal of all variants located outside refined locus, (2) all variants with a MAF ≥ 0.005 in publicly available Kaviar, gnomAD, 1000G, UK10K, GoNL datasets, and (3) that were shared between the four affected individuals. Abbreviations are as follows: Kaviar, Kaviar Genomic Variant database; gnomAD, The Genome Aggregation Database; 1000G, 1000 Genomes Project; UK10K, UK10K Rare Genetic Variants in Health and Disease; GoNL, Genomes of the Netherlands. One variant (G>T) is within the *GRHL2* gene (intronic) whereas the remaining two were intergenic.

To further delineate the PPCD4 locus in family C15, rare variants filtered from WGS data, including the c.20+544G>T variant in intron 1 of *GRHL2*, were genotyped and assessed for segregation in the extended pedigree by Sanger sequencing. Importantly, additional recombination events were identified in family C15 that further refined the PPCD4 locus to between chr8.hg38:101,411,163 and 109,214,442 excluding the two intergenic variants as candidates (Figure S1).

Interrogation of ENCODE data to identify potential enhancer and promoter regions of *GRHL2* revealed a cluster of transcription factor binding sites upstream of *GRHL2*, and spanning the 5′ UTR, first exon, and partial region of intron 1. The transcription factor binding prediction tools MatInspector and AliBaba 2.1 predict that the c.20+544G>T variant disrupts binding sites, leading to loss, or gain, of multiple transcription factors that are expressed in the corneal endothelium (Table 2 and Figure S1). Further analysis of c.20+544G>T in ENCODE data (Ensembl) identified this precise base location in intron 1 as a bivalent histone modification site, with histone H3 lysine 4 trimethylation (H3K4me3) and histone H3 lysine 27 trimethylation (H3K27me3) modifications in different cell types, which are associated with gene activation and repression, respectively^39^, ^40^ (Table 2 and Figure S2). In addition, this location marks a DNase I hypersensitive site and CTCF binding site, commonly associated with accessible chromatin and transcription factor binding and for forming local chromatin loops necessary for the tethering of promoters with associated regulatory elements, respectively^41^, ^42^ (Table 2 and Figure S2).

**Table 2:** Three *GRHL2* Intron 1 Variants Associated with PPCD4

							**Open Chromatin/Methylation Marks**		
**Coordinates (hg19)**	**Coordinates (hg38)**	**HGVS**	**CADD Score**	**Family**	**TF Gained**	**TF Lost**	**H1ESC**	**NHDF-AD**	**NHEK**
102,505,274	101,493,046	c.20+257delT	6.62	B4	POC/Zinc finger proteins, STAT6	EBF1	CTCF DNase1 H3K4me1	DNaseI H3K36me3H3K9ac	CTCF DNaseI H3K4me1 H3K9ac
102,505,150	101,492,922	c.20+133delA	13.23	B5	GLI3, ZBTBZA	ZNF354C	DNaseI H3K4me1 H3K4me3 H3K9ac	DNaseI H3K9ac	CTCF H3K4me1 H3K9ac
102,505,561	101,493,333	c.20+544G>T	10.76	C15 C23 C26 C33	ESRRA GLIS1 E2F3	SP1 NRF1 MYC E2F2 AHR	CTCF DNaseI H3K4me1	CTCF H3K4me1	CTCF DNaseI H3K4me3 H3K9ac

Three novel regulatory region variants in *GRHL2* identified in British and Czech families. Variants were located in intron 1 of *GRHL2,* identified by WGS (C15, C23, C33, C26) or targeted sequencing (B4, B5). All three variants are absent from public databases (Kaviar, gnomAD, 1000G, UK10K, GoNL datasets), have high CADD scores, are predicted to gain or lose binding sites for TFs expressed in corneal endothelium, and fall in peaks for open chromatin or methylation marks associated with gene regulation for different cell lines. Abbreviations are as follows: H1ESC, human embryonic stem cells; NHDF-AD, adult dermal fibroblasts; NHEK, normal human epidermal keratinocytes; CTCF, CCCTC-binding factor; DNaseI, deoxyribonuclease I; H3K9ac, H3 lysine 9 acetylation; H3K4me, H3 lysine4 monomethylation; H3K4me3, H3 lysine 4 trimethylation; H3K36me3, H3 lysine 36 trimethylation.

GRHL2 is a member of a highly conserved family of transcription factors, with an essential role during epithelial differentiation and suppression of EMT.^14^, ^43^ GRHL2 acts as a direct transcriptional repressor of *ZEB1*.^44^ Furthermore, GRHL2 is also thought to *trans*-activate *OVOL2* expression, forming a signaling network that regulates EMT and stabilizes epithelial specific gene expression.^45^ Given the role of *ZEB1* haploinsufficiency and potential inappropriate ectopic expression of *OVOL2* in the pathogenesis of PPCD, the c.20+544G>T variant in *GRHL2* represented an outstanding candidate disease-causing variant in family C15.

### Targeted Screening of *GRHL2* Regulatory Regions in Unsolved PPCD-Affected Families

**Figure 2:**
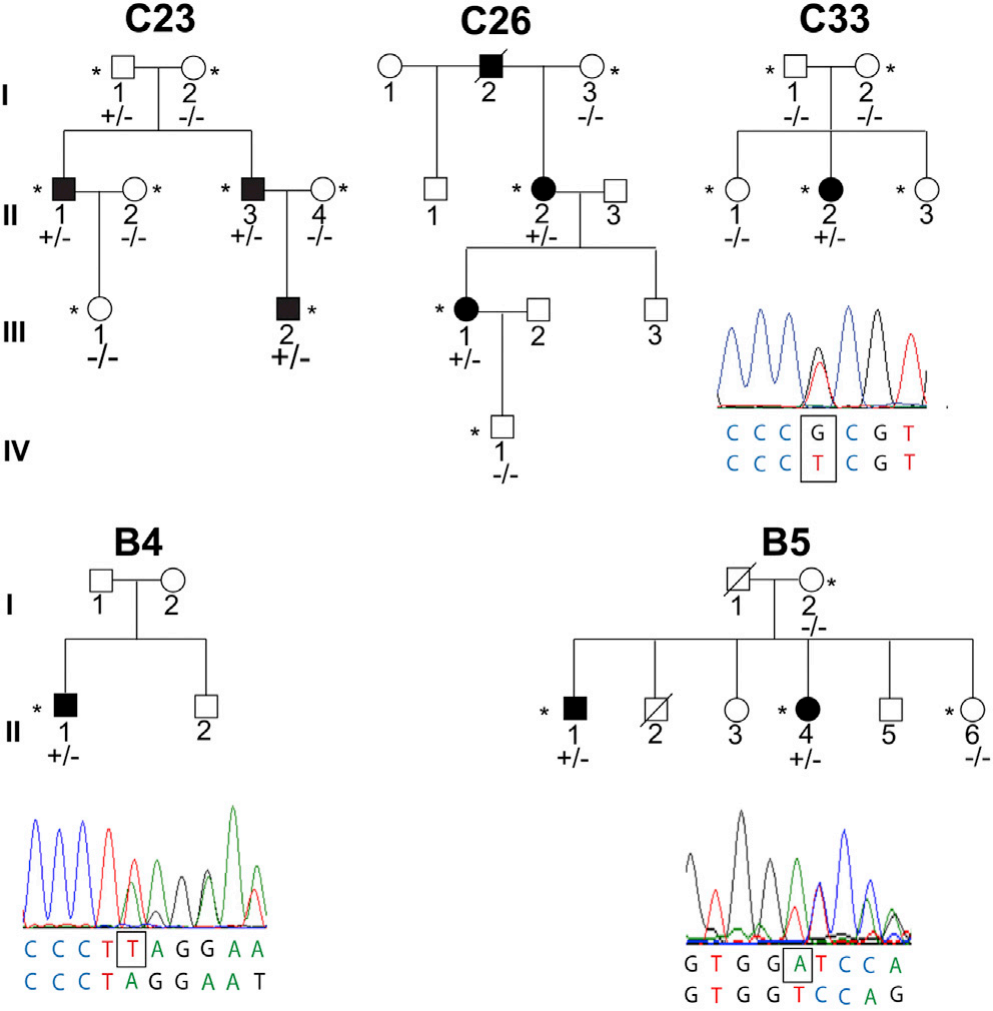
Additional PPCD-Affected Families with *GRHL2* Regulatory Region Mutations Presence of a *GRHL2* variant, shown in the electropherograms, is indicated by +/− in each family and absence by −/−. The heterozygous variant in intron 1 of *GRHL2*, c.20+544G>T, was found to segregate with disease in families C23 and C26 of Czech origin who share an ancestral haplotype with C15. The same mutation was identified in an affected individual in family C33 that occurred *de novo*. Two other mutations in intron 1 of *GRHL2* were identified; a 1-bp deletion, c.20+257delT (chr8.hg38:101,493,046delT), was identified in the proband in family B4, and a 1-bp deletion, c.20+133delA (chr8.hg38:101,492,922delA), in affected individuals in family B5. *GRHL2* variants are annotated according to transcript GenBank: NM_024915 and Ensembl ENST00000251808.7.

Given that the c.20+544G>T variant lies within a regulatory region of *GRHL2*, a 2,728-bp region encompassing the 5′ UTR, exon 1, and partial intron 1 of *GRHL2* containing predicted regulatory regions and transcription factor binding sites (Figure S2), was therefore PCR amplified and Sanger sequenced in unsolved PPCD cases. The same variant, c.20+544G>T, was identified in three additional families of Czech origin (C23, C26, and C33, Figure 2). None of the probands were knowingly related to the original pedigree or to each other. In two families (C23 and C26), the variant was shown to segregate with disease; however, in family C33 the proband was the only affected individual, and the variant was absent in both parents (Figure 2). Paternity testing^3^ confirmed the identity of the proband’s biological father, thereby suggesting that the variant occurred *de novo* in this individual.

To investigate potential ancestral haplotypes in families of Czech origin, rare variants identified in the WGS data from family C15 that refined the PPCD4 locus were genotyped by Sanger sequencing. The same mini-haplotype was identified in families C23 and C26, with an additional recombination event refining the haplotype (chr8.hg38:101,411,163–102,437,115), suggesting that the *GRHL2* variant in these families arose in a common ancestor (Figure S1). This analysis also confirmed the lack of an ancestral haplotype in family C33, further supporting the finding that this variant arose independently.

Screening the 2,728-bp *GRHL2* region in 19 genetically unsolved, unrelated PPCD-affected case subjects from the UK cohort identified two further variants. A single-nucleotide deletion c.20+257delT (chr8.hg38:101,493,046del; Figure 2) in intron 1 of *GRHL2* was identified in a proband from family B4 (Table 2). In family B5, a 1-bp deletion, also situated within the first intron of *GRHL2*, c.20+133delA (chr8.hg38:101,492,922del), was identified in the proband (II:4) and her affected brother (II:1) and was absent in her unaffected sister (II:6) (Figure 2 and Table 2). Both variants were absent from the control databases gnomAD, Kaviar, 1000G, GoNL, and UK10X (Figure 2 and Table 2).

To further verify the pathogenicity of the *GRHL2* variants (Table 2), a genomic region encompassing the Czech c.20+544G>T variant and the two other variants identified in individuals with PPCD, was Sanger sequenced in 210 Czech control samples (420 alleles). None of the PPCD-associated variants were detected in the control cohort. Interestingly, only a single heterozygous variant (rs548346355) was identified in a single individual in the control cohort, suggesting that this region is a highly conserved region. Similar to the variant identified in the Czech families, the c.20+257delT and c.20+133delA variants occur within regions rich in transcription factor binding sites, DNase I, CTCF sites, and histone modification domains identified by interrogating ENCODE data (Table 2 and Figure S3). All PPCD4-associated variants are predicted (MatInspector and AliBaba 2.1) to result in the gain, or loss, of binding of at least one transcription factor expressed in the corneal endothelium (Tables 2 and S1, Figure S3).

### Clinical Characterization of PPCD4

**Figure 3:**
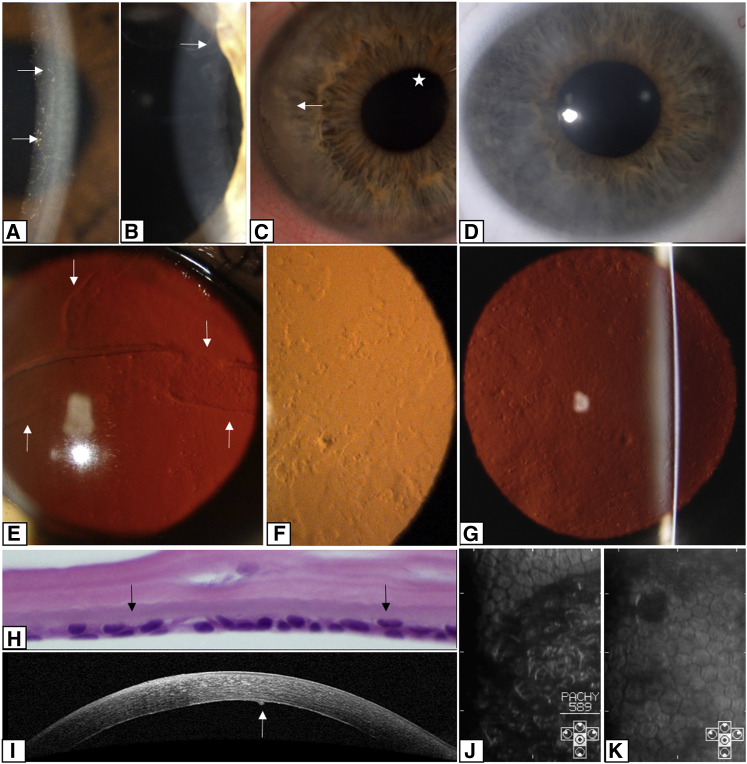
Corneal Disease Associated with Regulatory Region Mutations in *GRHL2* (A) Lines and vesicules (arrows) seen on oblique illumination in the left cornea of individual II:4 from family B5 (age 30 years). (B) A prominent posterior corneal line (arrow) seen on oblique broad-beam illumination of individual II:3 from family C23 (age 46 years). (C) Subepithelial calcium deposition (arrow) and corectopia (asterisk) in the right eye of individual VII:6 from family C15 (age 29 years). (D) Diffuse corneal stromal haze seen on direct illumination in individual III:2 from family C23 (age 11 years). (E–G) Retroillumination of the cornea to show bands (arrows) and vesicular and geographic shaped lesions. Shown are (E) right cornea of individual II:1 from family B4 (age 53 years), (F) left cornea of individual II:4 from family B5 (age 30 years) showing irregularity of reflex from scattered lesions at the posterior corneal surface, and (G) left cornea of individual VI:9 from family C15 (age 58 years). (H) Histological specimen of central corneal section from individual III:2 from family C23 (age 8.5 years) (H&E, magnification 600×); endothelial cells have formed a double layer (arrows). (I) SD-OCT cross-section of the right cornea of individual VI:9 from family C15 (age 58 years) shows increased reflectivity of the posterior corneal layers with a protrusion of Descemet membrane (arrow). (J and K) Specular microscopy images showing regional variation in the size and shape of the endothelial cells: (J) right eye of individual VIII:3 from family C15 (age 37 years) and (K) left eye of individual II:2 from family C33 (age 5.5 years). The dark areas correspond to areas displaced from the plane of specular reflection, presumably caused by protrusions and irregularities of the posterior corneal surface.

In this cohort, PPCD4 was found to display both inter- and intra-familial phenotypic variation. In the 27 affected individuals from families of Czech origin, harboring the same *GRHL2* mutation, 26 had typical corneal signs of PPCD, with an irregular posterior corneal surface and occasional opacities of variable size and shape clinically described as bands or geographic or vesicular lesions (Figures 3A, 3B, and 3G). The disease presented subjectively as blurred vision due to corneal edema in four individuals (Figure 3D). In two children, corneal edema and associated irritation of the eye was noted at 2 and 3 months after birth. Five individuals initially presented with a diagnosis of secondary glaucoma, either during a regular check-up, familial screening, or due to the development of corectopia (Figure 3C), prompting a visit to an eye specialist. Five individuals had low vision in one or both eyes since childhood and have not reported subsequent major changes of their visual function. One subject noticed decrease in visual acuity in the second decade of life. Nine individuals were asymptomatic at their last examination, and in one individual, information about the subjective onset was not available.

**Figure 4:**
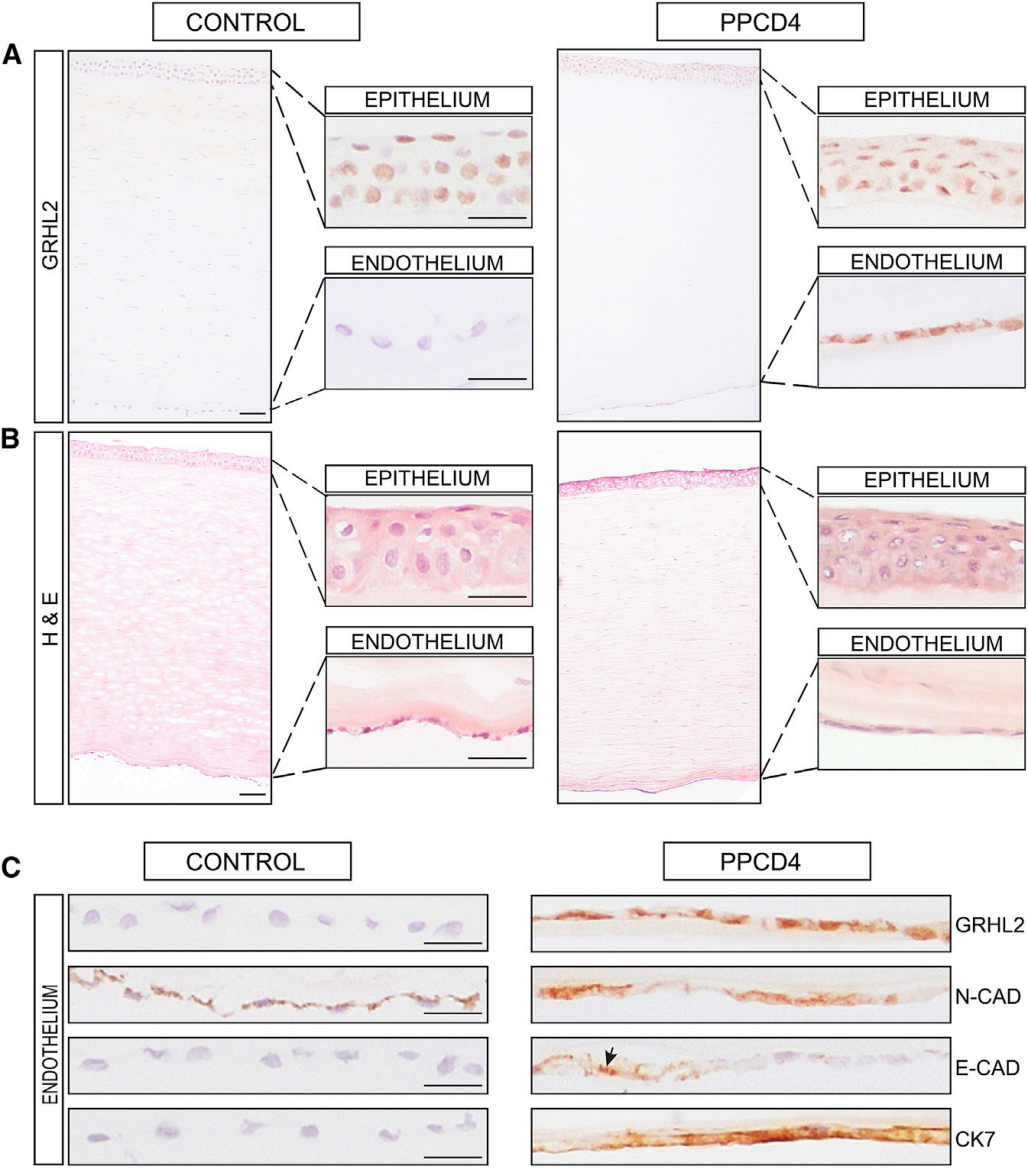
Immunohistochemistry of a PPCD4 Diseased Cornea Reveals GRHL2 Immunoreactivity and Cell State Transition in the Corneal Endothelium (A) Full thickness corneal tissue from control (left) and affected (right) individual with a c.20+544G>T *GRHL2* mutation (II:1 family C23) were stained with anti-GRHL2. Magnified images of the epithelium and endothelium are shown in insets. GRHL2 is detected in the nuclei of control and PPCD4 corneal epithelial cells but is absent in the control endothelium. In contrast, GRHL2 is also detected in the nuclei of the PPCD4 endothelial cells. (B) H&E staining showing integrity of full-thickness corneal sections for the diseased and control samples. (C) Magnified images of control and PPCD4 endothelial cells stained for GRHL2 and corneal epithelial and endothelial markers N-Cadherin (N-CAD), E-Cadherin (E-CAD), and Cytokeratin 7 (CK7). GRHL2 is localized in the nuclei in diseased endothelial cells. N-Cadherin was detected in control endothelial tissue and in the diseased endothelium. E-cadherin was negative in control endothelium but is expressed in the diseased endothelial tissue (arrowhead) with some areas of negative staining. CK7 was positive in the diseased endothelium and negative in the control sample. All sections were counterstained with Mayer’s hematoxylin to identify nuclei. Scale bar 50 μm.

At the last follow-up, best corrected visual acuity ranged from 1.0 bilaterally in five individuals to light perception in a 55-year-old male with secondary glaucoma and bullous keratopathy. Corneal transplantation was performed in 7 out of 27 (25.9%) individuals, and of these, 3 had bilateral surgery. The mean age of the first surgery was 34.9 ± 17.9 years (range 8.5 to 59 years). Glaucoma was diagnosed in seven individuals (25.9%), unilaterally in one male. The mean age of a diagnosis of glaucoma was 46.4 ± 17.1 years (range 20 to 63 years), but two subjects developed glaucoma after penetrating keratoplasty, and in these individuals, glaucoma may have been precipitated by surgery. Two subjects had an enucleation of a painful blind eye, one at the age of 25 years and the second at 70 years. Corectopia was noted in four eyes of three individuals and was associated with secondary glaucoma in all case subjects. Secondary corneal calcification (band keratopathy) developed bilaterally in two individuals (Figure 4C).

Specular microscopy and SD-OCT imaging documented a reduced endothelial cell density, with both normal and abnormal morphology and irregularities of the posterior corneal surface (Figures 3I–3K). In a 79-year-old individual, the disease status was unknown because the corneal periphery was obscured by age-related stromal haze. Although the corneal center was clear, the endothelial cell density count was 1,295 cells/mm^2^ in the right eye and 1,309 cells/mm^2^ in the left eye (normal range 2,400–2,600 cells/mm^2^).^46^ H&E staining of a full-thickness corneal sample (individual III:2, family C23) revealed an oedematous cornea with variation in endothelial cell size and shape and focal multilayering of the cells (Figure 3H).

The proband of B4 had an unusually prominent fold of Descemet membrane (Figure 3E). There was no family history of eye disease and the other family members were unavailable for examination.

Proband B5 (II:4) had markedly asymmetric disease with diffuse geographic endothelial changes restricted to her left eye (Figures 3A and 3F). She also had left amblyopia and a decompensated left exotropia. Intraocular pressures were normal in both eyes and there were no iris abnormalities. The endothelial cell density was markedly reduced in her affected eye (871 cells/mm^2^) compared to 3,165 cells/mm^2^ in her right eye. Her brother (II:1) carried the same *GRHL2* variant but had a significantly different phenotype. Although the endothelial cell count was lower than expected (1,900 cells/mm^2^ both eyes), there were no changes in cell morphology. Notably, numerous elevated Hassal-Henle bodies were present in the far periphery of the cornea.

### Expression of *GRHL2* in Healthy and Diseased Corneal Endothelium

Our interrogation of publicly available RNA-seq data from healthy adult and fetal human corneal endothelial tissue revealed no evidence of *GRHL2* expression^47^ (Figure S4A). Examination of additional publicly available RNA-seq data also confirmed lack of *GRHL2* expression in corneal stromal cells, whereas high levels of expression were detected in the corneal epithelium^48^ (Figure S4A). We therefore further defined corneal expression of *GRHL2* in cultured cells by RT-PCR and the distribution of GRHL2 in corneal tissue by immunohistochemistry (IHC).

*GRHL2* expression was detected in cultured human corneal epithelial cells derived from limbal epithelial stem cells and in a spontaneously immortalized human corneal epithelial cell line with progenitor-like characteristics, but was absent in corneal endothelial tissue, an immortalized cell line of human corneal endothelial origin, and stromal fibroblasts by RT-PCR (Figure S4B).

*GRHL2* encodes a transcription factor that is a direct transcriptional repressor of *ZEB1*.^49^ Given this role, in addition to the lack of *GRHL2* expression in the corneal endothelium, we hypothesized that the putative regulatory mutations could lead to inappropriate transcriptional activation and ectopic expression of *GRHL2* in the corneal endothelium, similar to the mechanism we proposed for the variants in *OVOL2*.^2^ To explore this hypothesis further, a full-thickness corneal sample from individual II:1 from family C23, with the *GRHL2* c.20+544G>T variant, was analyzed by IHC and compared to control tissue. First, we tested for presence of GRHL2 in different cell layers. GRHL2 was detected in the nuclei of epithelial cells in control tissue, consistent with its role as a transcription factor, and was absent from the stroma and endothelium, concordant with the transcriptomic data (Figure 4A). Strikingly, in the diseased cornea, endothelial cell nuclei were positive for GRHL2, suggesting that the c.20+544G>T *GRHL2* variant induces ectopic expression of *GRHL2* resulting in detection of GRHL2 protein in the corneal endothelium.

Differences in the levels of epithelial, mesenchymal, and endothelial markers were also observed between the diseased and control endothelial cells (Figure 4C). N-Cadherin, which is normally detected in corneal endothelial and epithelium cells,^50^, ^51^ was detected in the control endothelium and the diseased tissue (Figure 4C). In contrast, E-Cadherin, a component of adherens junctions and marker of epithelial cell status, is not detected in healthy corneal endothelium;^47^, ^50^ however, regions of positive staining for E-Cadherin were evident in the PPCD4 endothelium, which was negative in the control, indicating that the cells had diverged from their normal identity (Figure 4C). This upregulation of E-Cadherin is consistent with cells undergoing MET.

Previous IHC studies of PPCD1 and PPCD3 samples have shown inappropriate positive staining for keratins in diseased tissue.^5^ CK7, a marker of corneal epithelial cells, was positive in the diseased endothelium and negative in the control sample (Figure 4C). Collectively, these data indicate that the PPCD4 endothelial cells were in transition to an epithelial-like cell type or had already diverged. We hypothesize that this diseased cell state transition is due to ectopic expression of *GRHL2* in the corneal endothelium, induced by the c.20+544G>T variant.

### Promoter Mutations Result in Increased Expression of *GRHL2*

**Figure 5:**
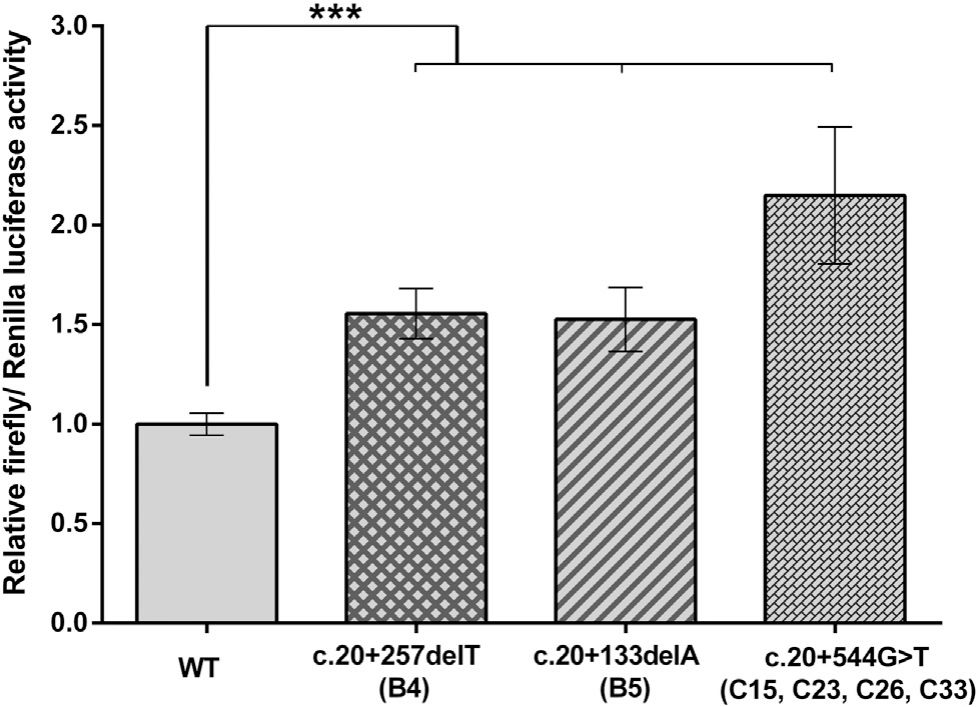
Intron 1 of *GRHL2* Is Transcriptionally Active and PPCD4 Mutations Cause Increased Promoter Activity *In Vitro* A dual-luciferase reporter assay was used to determine whether intron 1 of *GRHL2* had promoter activity and the effect of PPCD4 mutations. HEK293 cells were co-transfected with pRL-CMV (*Renilla* luciferase) and pGL3-basic (firefly luciferase) containing 2,728 bp of the wild-type or respective *GRHL2* promoter sequence mutants. Wild-type activity was normalized to 1, and the relative luciferase activity of all mutants was expressed with respect to the wild-type. All PPCD4-associated *GRHL2* variants significantly increased the relative luciferase activity. Data represent a minimum of three independent biological replicates in triplicate. Error bars represent ±1 SD. p values were calculated by one-way ANOVA (^∗∗∗^p ≤ 0.001).

Given the striking ectopic detection of GRHL2 in diseased PPCD4 corneal endothelial cells (Figure 4), and that *in silico* analysis of all three PPCD4 variants identified are predicted to alter transcriptional activity (Table 2 and Figure S3), we experimentally tested how each of these variants alter *GRHL2* promoter activity *in vitro*. A 2,728-bp fragment encompassing the position of all three variants and predicted surrounding regulatory regions was cloned into a promoter-less firefly luciferase reporter vector. The PPCD4-associated variants were independently introduced by site-directed mutagenesis. HEK293 cells were co-transfected with each of the *GRHL2* promoter constructs to test promoter activity, in combination with *Renilla* luciferase for normalization purposes. The wild-type *GRHL2* construct was an active promoter region driving expression of firefly luciferase (Figure 5). Each of the three *GRHL2* PPCD4-associated mutations were found to significantly (p ≤ 0.001) increase the promoter activity of the region compared to the wild-type sequence (Figure 5).

## Discussion

In this study we identified a locus for autosomal-dominant PPCD, PPCD4, on chromosome 8q22.3–q24.12. WGS revealed three unique non-coding variants within the linked region in the index family (C15), one of which, c.20+544G>T, mapped within a potential regulatory region of *GRHL2*. Additional recombination events were identified by genotyping in the extended family, that refined the PPCD4 locus (chr8.hg38:101,411,163–109,214,442) and excluded two of the variants, leaving c.20+544G>T as the outstanding candidate disease-causing variant.

The same variant was found in two additional unsolved PPCD-affected families of Czech origin that shared an ancestral haplotype with family C15. A *de novo* occurrence of this variant was identified in another PPCD-affected individual, suggesting that this is a recurrent mutation. Two further unique variants were found in intron 1 of *GRHL2* (c.20+257delT and c.20+133delA) in additional unrelated individuals affected with PPCD.

All three *GRHL2* mutations were located in a conserved uncharacterized regulatory region. We hypothesize that the mechanism of disease is similar to the mechanism we and others proposed for *OVOL2* promoter mutations that cause PPCD1, whereby mutations lead to an overactive promoter that drives inappropriate ectopic expression of *OVOL2* in the corneal endothelium.^2^, ^12^ To understand further how the promoter mutations affect expression of *GRHL2* in corneal endothelial cells and contribute to the pathogenesis of PPCD4, we performed IHC on a corneal sample from a PPCD4-affected individual. *GRHL2* is not normally expressed in the corneal endothelium *in vivo*; however, in PPCD4-diseased corneal tissue, we detect GRHL2 in the corneal “endothelium” supporting the hypothesis that the PPCD4 variants result in inappropriate activation and ectopic expression of *GRHL2*. Furthermore, we demonstrate that this putative regulatory region of *GRHL2* is transcriptionally active and that all PPCD4-associated variants significantly increased *GRHL2* promoter activity compared to wild-type.

Interestingly, mutations leading to presumed haploinsufficiency of *GRHL2* cause autosomal-dominant non-syndromic hearing impairment (DNFA28 [MIM: 608641])^52^, ^53^, ^54^ and homozygous missense mutations have been associated with autosomal-recessive ectodermal dysplasia syndrome with hearing loss (ECTDS [MIM: 616029]).^55^ None of the PPCD-affected individuals with *GRHL2* promoter mutations in our study reported hearing loss or other features of ectodermal dysplasia syndrome. This is not unexpected given that neither of the previously described *GRHL2*-associated disease mechanisms are predicted to result in the aberrant upregulation and ectoptic expression of *GRHL2*.

In addition to *GRHL2* in the PPCD4 corneal “endothelium,” we also detected E-Cadherin and Cytokeratin 7, consistent with cellular state transition as the mechanism of disease for PPCD4, through the MET pathway. This is also consistent with the abnormal “endothelial” cell morphology detected in PPCD4-affected case subjects using specular microscopy and examination of histological sections.

**Figure 6:**
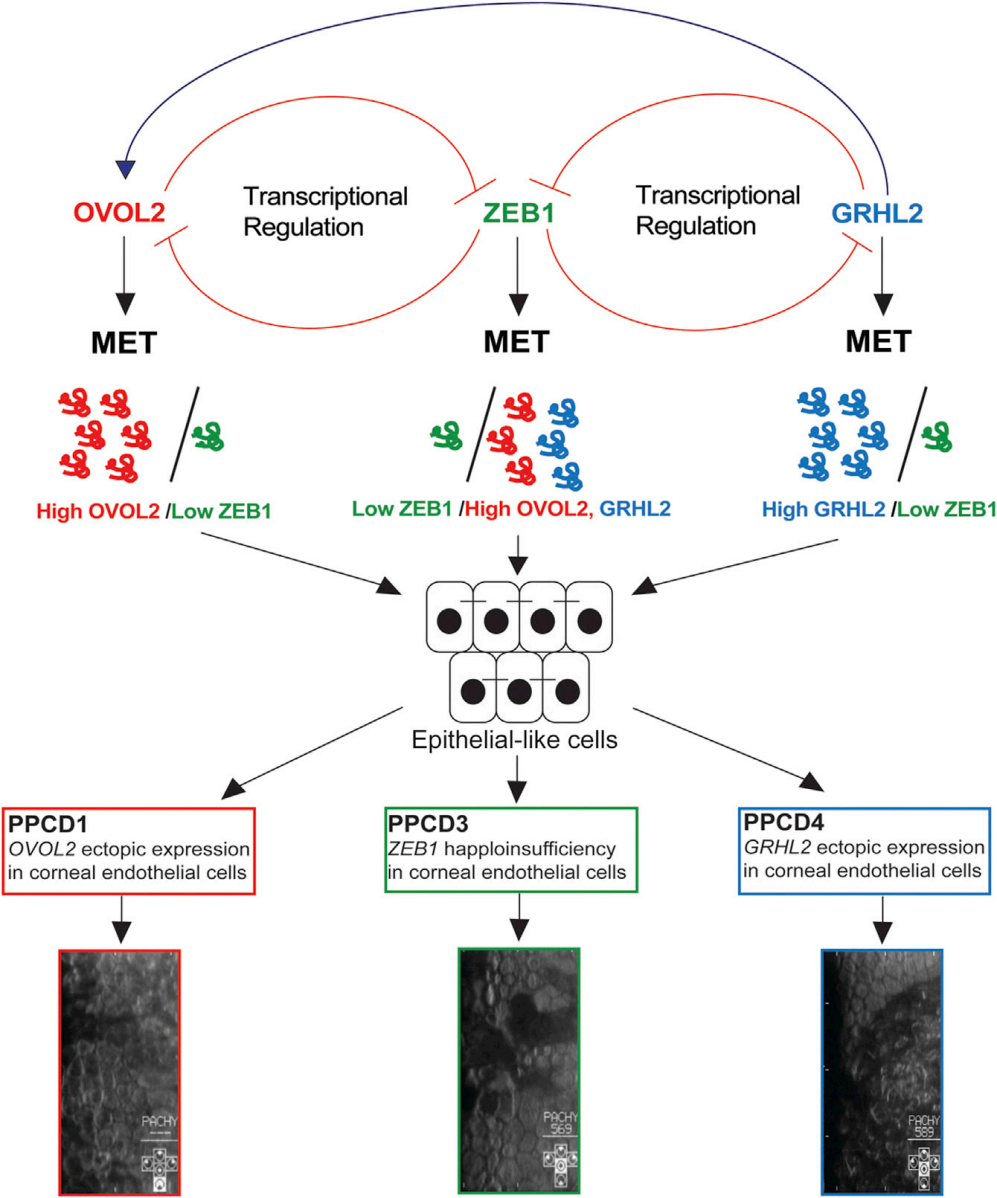
Schematic of Proposed Model of PPCD Pathogenicity Epithelial-to-mesenchymal transition (EMT) and mesenchymal-to-epithelial transition (MET) are mediated by a precise regulation of transcription factors. ZEB1 promotes EMT and maintains the normal state of human corneal endothelial cells. OVOL2 and GRHL2 are direct transcriptional repressors of *ZEB1*. ZEB1 can also repress the expression of both of these genes, and this mechanism may maintain the cellular state of healthy corneal endothelial cells. In PPCD, mutations that cause haploinsufficiency and reduced expression of *ZEB1* and promoter mutations that drive ectopic expression of *GRHL2* or *OVOL2* in corneal endothelial cells are the proposed mechanisms that result in cell state transition through the MET pathway. GRHL2 activates the expression of *OVOL2*, so ectopic expression is predicted to also result in ectopic expression of OVOL2 and repression of ZEB1, driving corneal endothelium cells to transition to epithelial-like cells, presenting as stratified and irregularly shaped cells.

GRHL2 has an important role in epithelial morphogenesis through *trans*-activation of genes required for the formation of the apical junctional complex and repression of EMT. In duct cells of the kidney, GRHL2 regulates lumen expansion and epithelial barrier formation by *trans*-activating *OVOL2* expression, which in turn activates the expression of E-Cadherin, claudin 4 (epidermal tight junctions), and Rab25 (apical trafficking).^56^ OVOL2 maintains the transcriptional program of human corneal epithelium cells by repressing expression of mesenchymal genes such as *ZEB1*.^14^, ^49^ Similarly, GRHL2 is known to be a direct transcriptional repressor of *ZEB1*.^44^ Importantly, haploinsufficiency of *ZEB1* causes PPCD3, and ectopic expression of *OVOL2* in the corneal endothelium caused by promoter mutations, leading to repression of *ZEB1* transcription, is the mechanism proposed for PPCD1^2^, ^10^, ^12^, ^21^ (Figure 6). Therefore, we propose that ZEB1, OVOL2, and GRHL2 form a finely balanced mutually inhibitory EMT/MET pathway that controls specific cell characteristics and intermediate cell states^13^, ^14^, ^49^ (Figure 6).

In support of this proposed mechanism, a transcriptomic profile of PPCD corneal endothelial cells derived from a subject with PPCD3 harboring a pathogenic *ZEB1* mutation and a PPCD-affected individual with unknown molecular cause, with no potentially pathogenic variant detected in *ZEB1* or the *OVOL2* promoter using the methods employed, revealed a significant decrease in *ZEB1* expression compared to controls.^21^ Furthermore, transcriptomic data of the corneal endothelium of an individual with PPCD of undefined genetic cause identified *GRHL2* as the most differentially expressed gene (upregulated) compared to controls.^21^

Collectively, these data support the disease mechanism of ectopic expression of *GRHL2* in PPCD4 endothelial cells as a result of mutations in a regulatory region and that MET is a convergent pathogenic mechanism leading to intermediate cell states and dysfunction of the endothelial barrier and disease in PPCD (Figure 6).

## Accession Numbers

*GRHL2* variants associated with disease have been submitted to ClinVar.

## Web Resources

1000 Genomes, http://www.internationalgenome.org/
Alibaba 2.1, http://gene-regulation.com/pub/programs/alibaba2/index.html
ClinVar, https://www.ncbi.nlm.nih.gov/clinvar/
dbSNP, https://www.ncbi.nlm.nih.gov/projects/SNP/
ENCODE, https://genome.ucsc.edu/ENCODE/
Ensembl Genome Browser, http://www.ensembl.org/index.html
ExAC Browser, http://exac.broadinstitute.org/
ExomeDepth, http://cran.r-project.org/web/packages/ExomeDepth/index.html
GATK, https://software.broadinstitute.org/gatk/
gnomAD Browser, http://gnomad.broadinstitute.org/
GoNL (Genomes of the Netherlands), http://www.nlgenome.nl/search/
IGV, http://www.broadinstitute.org/igv/
Kaviar Browser, http://db.systemsbiology.net/kaviar/
MatInspector, http://www.genomatix.de/index.html
NCBI Genome build GRCh38, https://www.ncbi.nlm.nih.gov/assembly?term=GRCh38&cmd=DetailsSearch
NHLBI Exome Sequencing Project (ESP) Exome Variant Server, http://evs.gs.washington.edu/EVS/
Novoalign, http://www.novocraft.com/products/novoalign/
OMIM, http://www.omim.org/
RefSeq, http://www.ncbi.nlm.nih.gov/RefSeq
SAMtools, http://www.htslib.org/
SeattleSeq Annotation, http://snp.gs.washington.edu/SeattleSeqAnnotation150/
Sequence Variant Nomenclature, http://varnomen.hgvs.org/
UCSC Genome Browser, http://genome.ucsc.edu
UK10K Consortium, http://www.uk10k.org/
Variant Effect Predictor, http://useast.ensembl.org/Homo_sapiens/Tools/VEP

## Supplemental Data

Document S1. Figures S1–S4 and Table S1

Document S2. Article plus Supplemental Data
