# Supplementary material for: Ectopic GRHL2 Expression Due to Non-coding Mutations Promotes Cell State Transition and Causes Posterior Polymorphous Corneal Dystrophy 4
Source: Am J Hum Genet. 2018 Mar 1;102(3):447–59. doi: 10.1016/j.ajhg.2018.02.002 (PMC5985340; doi:10.1016/j.ajhg.2018.02.002)
Supplement: Document S1. Figures S1–S4 and Table S1 [file mmc1.pdf]

The American Journal of Human Genetics, Volume *102*

Supplemental Data

Ectopic *GRHL2* Expression Due to Non-coding Mutations Promotes Cell State Transition and Causes Posterior Polymorphous Corneal Dystrophy 4

Petra Liskova, Lubica Dudakova, Cerys J. Evans, Karla E. Rojas Lopez, Nikolas Pontikos, Dimitra Athanasiou, Hodan Jama, Josef Sach, Pavlina Skalicka, Viktor Stranecky, Stanislav Kmoch, Caroline Thaung, Martin Filipec, Michael E. Cheetham, Alice E. Davidson, Stephen J. Tuft, and Alison J. Hardcastle

Supplemental Data

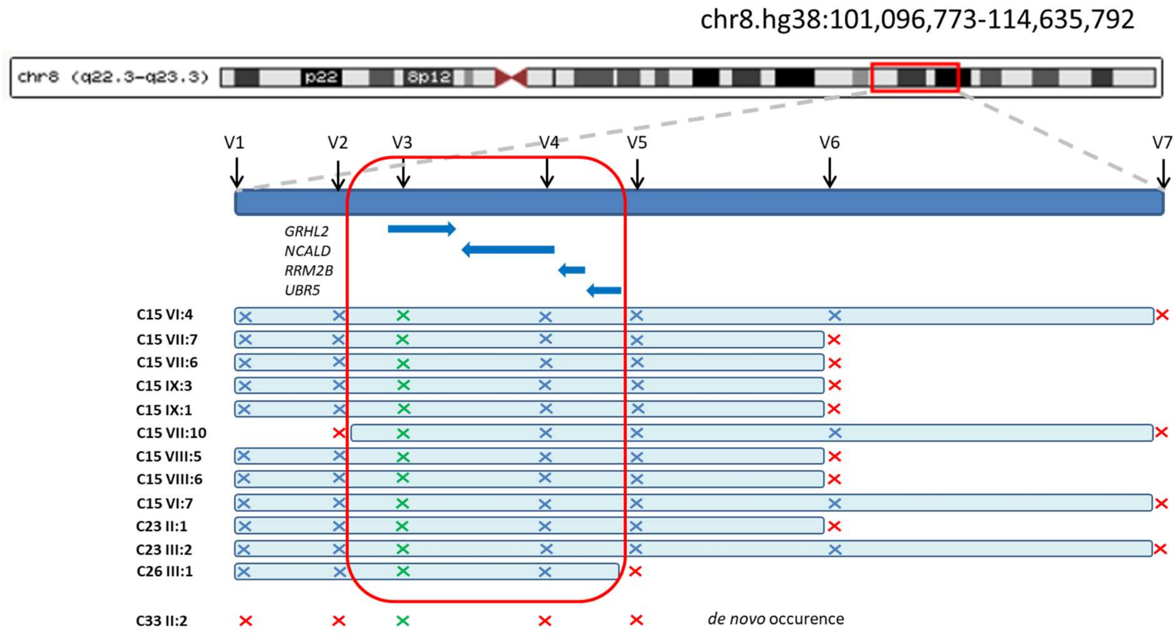

	V1	V2	V3	V4	V5	V6	V7
**Position (Build GRCh38)**	Chr8:101,096,773	Chr8:101,411,163	Chr8:101,493,333	Chr8:101,546,909	Chr8:102,437,115	Chr8:109,214,442	Chr8:114,635,792
**Variant**	G>A	T>C	G>T	A>G	G>A	AAAACA>TT	A>T
**dbSNP**	rs554736280	rs564548712	none	rs145339755	rs72683010	rs71303458	none
**MAF in 1000G**	A: 0.080% (4/5008)	C: 0.060% (3/5008)	unique	G: 0.781% (17/2178)	A: 0.120% (6/5008)	N/A	unique

Figure S1. Ancestral mini-haplotype of 3 families of Czech origin
Rare variants V1, V2, V4-V7 (see table at bottom of schematic haplotype) selected within the PPCD4 linked region (filled blue rectangles) were genotyped in individuals from families C15, C23 and C26 with the same *GRHL2* mutation (V3, green cross). Recombination event (red cross) were identified in family C15 that refined the locus and associated mini-haplotype. The same mini-haplotype was identified in families C23 and C26, with an additional recombination events, that suggests the *GRHL2* mutation in these families arose in a common ancestor. This ancestral mini-haplotype was absent in affected individual II:2 from family C33, who had the same *GRHL2* mutation that occurred *de novo*.

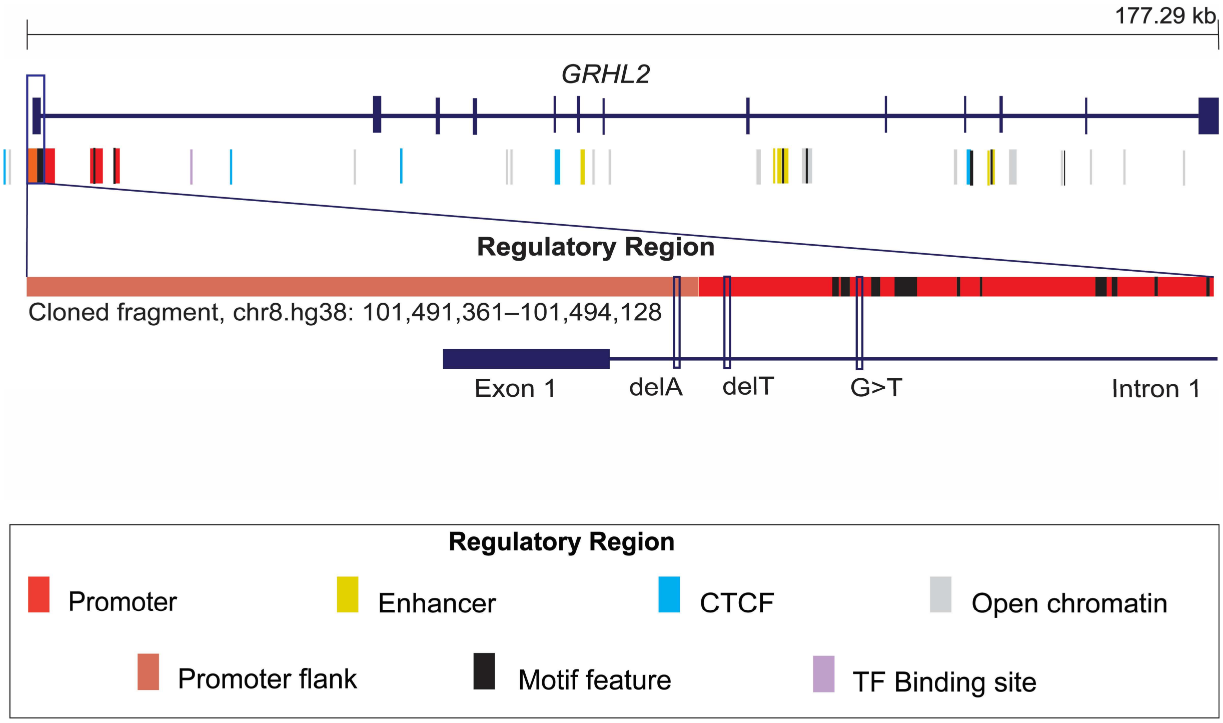

Figure S2. Schematic representation of the regulatory region surrounding the three *GRHL2* promoter mutations

GRHL2 gene structure showing regulatory element annotations from the Ensembl Regulatory Build, colored by feature. Zoom-in view of the intron 1 region encompassing all 3 mutations showing they fall within a predicted promoter region. The image is extracted from Ensembl v90 using GRCh38 assembly and GENCODE v22 gene annotations. G>T represents c.20+544G>T, delT represents c.20+257delT, delA represents c.20+133delA. Mutations are annotated in accordance with the *GRHL2* cDNA sequence (Ensembl: ENST00000251808.7). Red bar represents the regulatory region (2,728 bp) sequenced in unsolved PPCD patients and cloned for the luciferase assay.

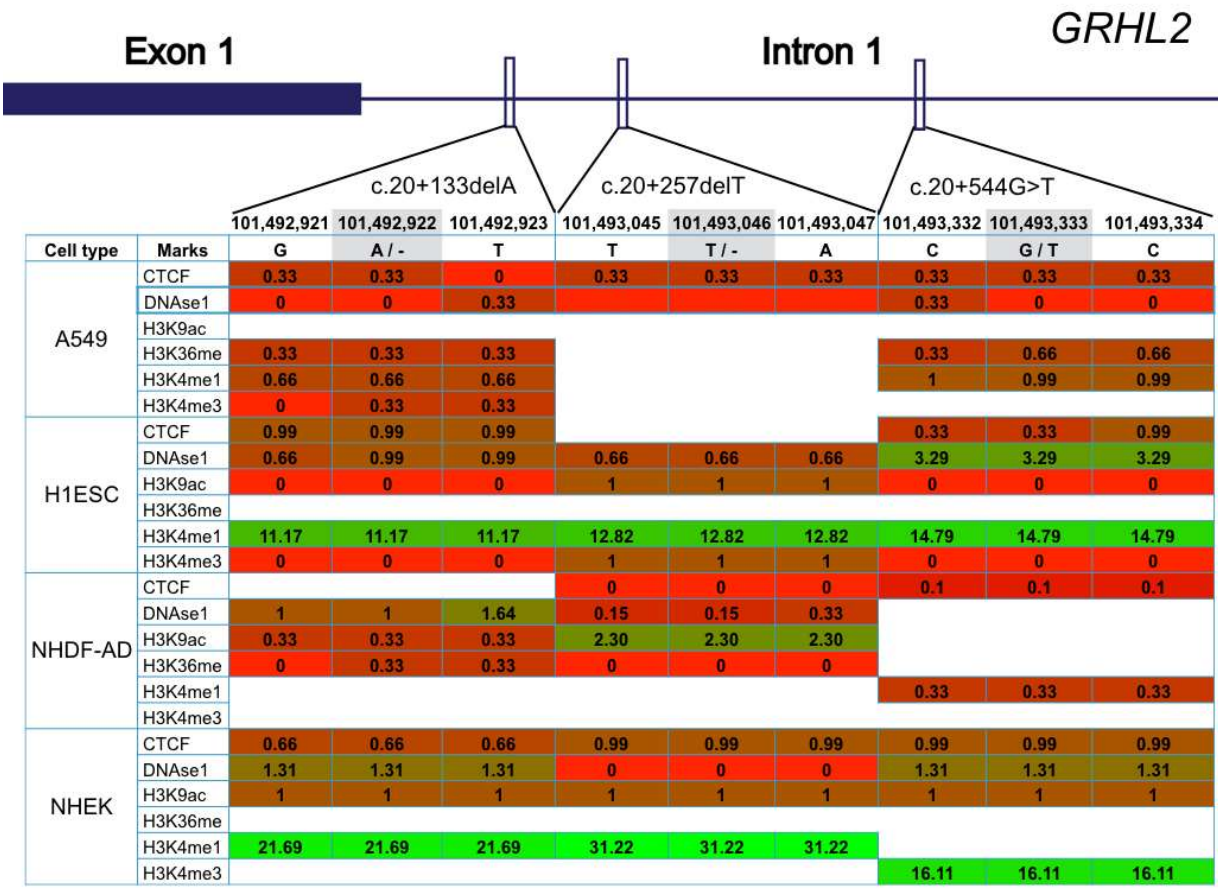

Cell type	Marks	101,492,921	101,492,922	101,492,923	101,493,045	101,493,046	101,493,047	101,493,332	101,493,333	101,493,334
		G	A / -	T	T	T / -	A	C	G / T	C
A549	CTCF	0.33	0.33	0	0.33	0.33	0.33	0.33	0.33	0.33
	DNAse1	0	0	0.33				0.33	0	0
	H3K9ac									
	H3K36me	0.33	0.33	0.33				0.33	0.66	0.66
	H3K4me1	0.66	0.66	0.66				1	0.99	0.99
	H3K4me3	0	0.33	0.33						
H1ESC	CTCF	0.99	0.99	0.99				0.33	0.33	0.99
	DNAse1	0.66	0.99	0.99	0.66	0.66	0.66	3.29	3.29	3.29
	H3K9ac	0	0	0	1	1	1	0	0	0
	H3K36me									
	H3K4me1	11.17	11.17	11.17	12.82	12.82	12.82	14.79	14.79	14.79
	H3K4me3	0	0	0	1	1	1	0	0	0
NHDF-AD	CTCF				0	0	0	0.1	0.1	0.1
	DNAse1	1	1	1.64	0.15	0.15	0.33			
	H3K9ac	0.33	0.33	0.33	2.30	2.30	2.30			
	H3K36me	0	0.33	0.33	0	0	0			
	H3K4me1							0.33	0.33	0.33
	H3K4me3									
NHEK	CTCF	0.66	0.66	0.66	0.99	0.99	0.99	0.99	0.99	0.99
	DNAse1	1.31	1.31	1.31	0	0	0	1.31	1.31	1.31
	H3K9ac	1	1	1	1	1	1	1	1	1
	H3K36me									
	H3K4me1	21.69	21.69	21.69	31.22	31.22	31.22			
	H3K4me3							16.11	16.11	16.11

Figure S3. Promoter features at the positions of the three intron 1 *GRHL2* mutations Zoomed-in view of promoter features for each of the 3 mutation positions within intron 1 of *GRHL2*. PCHi-C data from 4 cell types (A549 - epithelial lung carcinoma; H1ES - human embryonic stem cells; NHDF-AD - adult dermal fibroblasts; NHEK - normal human epidermal keratinocytes), and chromatin features based on ChromHMM segmentations of BLUEPRINT histone modification data are shown. PCHi-C data displays features such as DNase1 or CTCF binding sites. ChromHMM histone states for: H3 lysine 4 trimethylation (H3K4me3), H3 lysine4monomethylation (H3K4me1), H3 lysine 36 trimethylation (H3K36me3) for actively transcribed regions are shown. H3 lysine 9 acetylation is associated with promoter regions. The image is based on Ensembl v90 using GRCh38 assembly. Mutations are annotated in accordance with the *GRHL2* cDNA sequence (Ensembl: ENST00000251808.7). Brightness of colors (red and green, brighter = stronger) and numbers in boxes represent scores of relative strength of features.

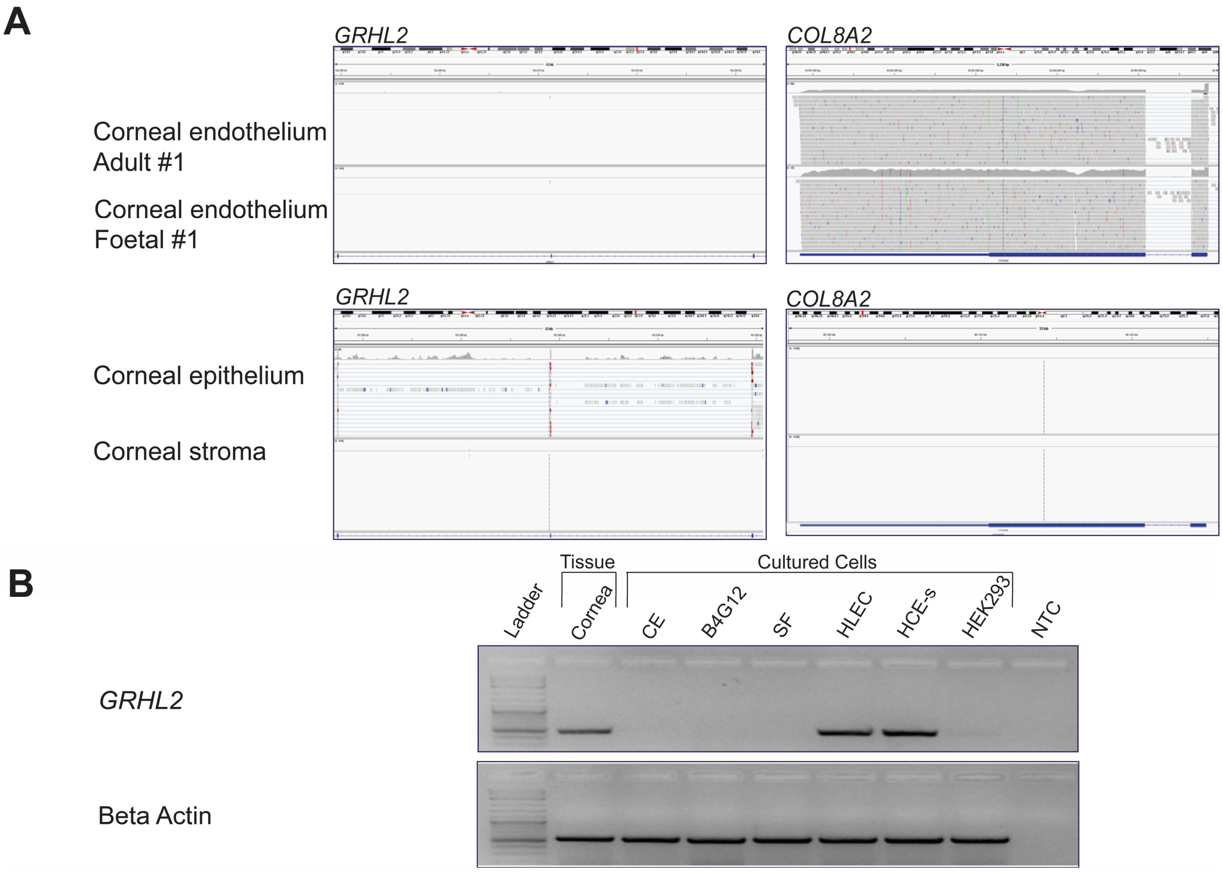

Figure S4. Expression of *GRHL2* in different layers of the human cornea
(A) Interrogation of RNA-seq data derived from adult and fetal human corneal endothelial samples revealed no evidence of *GRHL2* expression. *COL8A2* is highly expressed in corneal endothelium. *GRHL2* is expressed only in the corneal epithelium and not expressed in stroma. **(B)** RT-PCR of corneal tissue and cell types indicates *GRHL2* is expressed in full thickness cornea, human corneal epithelial cells (HCE-s), human limbal epithelial cells (HLEC). No expression was detected in cultured endothelial cells (CE and B4G12) or stromal cells (SF). NTC = no template control.

Table S1. ***In silico*** **predictions of the consequence of** ***GRHL2*** **variants on transcription factor binding**

AliBaba[1] 2.1 predicts transcription factor binding sites in an unknown DNA sequence utilizing the TRANSFAC public database. MatInspector[2] utilizes a large library of matrix descriptions for transcription factor binding sites to locate matches in DNA sequences. *RKPM values also encompass flanking transcripts. All 3 mutations were predicted to alter transcription factor binding (loss and gain).

**Transcription factor**	**C15, C23, C26, C33 c.20+544G>T**	**B4 c.20+257delT**	**B5 c.20+133delA**	**Expression in endothelium (RKPM)**	
				**Adult**	**Fetal**
SP1	Site lost[1]			12.52	17.53
NRF1*	4 sites lost[2]			9.31	12.78
ESRRA*	Site gained[2]			41.52	8.74
GLIS1	Site gained[2]			42.75	3.19
AHR	Site lost[2]			32.84	28.87
MYC	Site lost[2]			25.00	5.98
E2F2	Site lost[2]			0.07	1.44
E2F3	Site gained[2]			0.17	0.02
EBF1		Site lost[2]		0.05	5.98
POZ/zinc finger proteins	Site lost[2]	Site gained[2]		N/A	N/A
STAT6		Site gained[2]		27.53	21.94
ZNF354C			Site lost[2]	3.53	5.61
GLI3			Site gained[2]	20.15	14.68
ZBTB7A			Site gained[2]	17.91	7.01
